# First record of a tandem-repeat region within the mitochondrial genome of *Clonorchis sinensis* using a long-read sequencing approach

**DOI:** 10.1371/journal.pntd.0008552

**Published:** 2020-08-26

**Authors:** Liina Kinkar, Neil D. Young, Woon-Mok Sohn, Andreas J. Stroehlein, Pasi K. Korhonen, Robin B. Gasser

**Affiliations:** 1 Department of Veterinary Biosciences, Melbourne Veterinary School, Faculty of Veterinary and Agricultural Sciences, The University of Melbourne, Parkville, Victoria, Australia; 2 Department of Parasitology and Tropical Medicine, and Institute of Health Sciences, Gyeongsang National University College of Medicine, Jinju, Korea; University of Oxford, UNITED KINGDOM

## Abstract

**Background:**

Mitochondrial genomes provide useful genetic markers for systematic and population genetic studies of parasitic helminths. Although many such genome sequences have been published and deposited in public databases, there is evidence that some of them are incomplete relating to an inability of conventional techniques to reliably sequence non-coding (repetitive) regions. In the present study, we characterise the complete mitochondrial genome—including the long, non-coding region—of the carcinogenic Chinese liver fluke, *Clonorchis sinensis*, using long-read sequencing.

**Methods:**

The mitochondrial genome was sequenced from total high molecular-weight genomic DNA isolated from a pool of 100 adult worms of *C*. *sinensis* using the MinION sequencing platform (Oxford Nanopore Technologies), and assembled and annotated using an informatic approach.

**Results:**

From > 93,500 long-reads, we assembled a 18,304 bp-mitochondrial genome for *C*. *sinensis*. Within this genome we identified a novel non-coding region of 4,549 bp containing six tandem-repetitive units of 719–809 bp each. Given that genomic DNA from pooled worms was used for sequencing, some variability in length/sequence in this tandem-repetitive region was detectable, reflecting population variation.

**Conclusions:**

For *C*. *sinensis*, we report the complete mitochondrial genome, which includes a long (> 4.5 kb) tandem-repetitive region. The discovery of this non-coding region using a nanopore-sequencing/informatic approach now paves the way to investigating the nature and extent of length/sequence variation in this region within and among individual worms, both within and among *C*. *sinensis* populations, and to exploring whether this region has a functional role in the regulation of replication and transcription, akin to the mitochondrial control region in mammals. Although applied to *C*. *sinensis*, the technological approach established here should be broadly applicable to characterise complex tandem-repetitive or homo-polymeric regions in the mitochondrial genomes of a wide range of taxa.

## Author summary

In the present study, we characterised the complete mitochondrial genome of *Clonorchis sinensis*—a carcinogenic liver fluke. To do this, we sequenced from total genomic DNA from multiple adult worms using a new method (Oxford Nanopore technology) to obtain data for long stretches of DNA, and then assembled these data to construct a mitochondrial genome of 18,304 bp, containing a > 4.5 kb-long tandem-repetitive region—not previously detected in this species. The results demonstrate that this method is effective at sequencing long and complex non-coding elements—not achievable using conventional techniques. The discovery of this long tandem-repetitive region in *C*. *sinensis* provides an opportunity to now explore its origin(s) and length/sequence diversity in populations of this species, and also to characterise its function(s). The technological approach employed here should have broad applicability to characterise previously-elusive non-coding mitochondrial genomic regions in a wide range of taxa.

## Introduction

Substantial progress in nuclear and mitochondrial genomics has been made over the last two decades through the use of DNA sequencing methods [1]. This progress is starting to have a major positive impact in many areas of parasitology, both fundamental and applied. For instance, exploring the mitochondrial genomes has enabled systematic (taxonomic and phylogenetic) and population genetic investigations of helminths (flatworms and roundworms) [2–6]. Such genomes provide a rich source of markers for such investigations and are particularly applicable to systematic investigations of species of flatworms (platyhelminths) [7], because the mitochondrial genes are usually considerably less variable in sequence than for many roundworm (nematode) species [8–11]. Thus, there have been numerous studies of members of the classes Trematoda and Cestoda [7, 12–15].

Seminal work on mitochondrial genomes was conducted using PCR-based cloning combined with conventional (Sanger) sequencing (e.g., [7, 16]). Subsequently, high throughput sequencing (e.g., 454 and Illumina) became the approach of choice, allowing sequencing from small amounts of genomic DNA at reduced cost and time [1]. With the advent of ‘short-read’ sequencing (e.g., Illumina) came the confidence that sequencing at high coverage in a high throughput manner would readily allow the sequencing and assembly of complete mitochondrial genomes, because of their relatively small size (~ 14 kb ± 1 kb in flatworms; [14]). However, there have been challenges with sequencing through tandem-repetitive elements and regions with a biased nucleotide composition using Sanger and short-read technologies [16–18], and little attention has been paid to the impact of these issues.

Indeed, recently, when we explored mitochondrial genomes of parasitic flatworms of the genus *Echinococcus*, we noticed a gap of > 1 kb between the 3′-end of the *nad*5 gene and the 5′-end of the *cox*3 gene in *E*. *granulosus* genotype G1 [19]. Despite our efforts using a PCR-based sequencing strategy, we were not able to sequence this gap. However, employing a single molecule, real-time (SMRT) sequencing technology, we obtained long sequence reads that bridged the entire gap, allowing us to characterise a 4,417 bp-long tandem-repetitive region consisting of ten near-identical repeat units (441–445 bp), each harbouring a 184 bp non-coding region and flanking regions [17]. Although three mitochondrial genomes for *E*. *granulosus* genotype G1 had been published and/or deposited in public gene databases (including GenBank), closing this gap allowed us to define (what we considered to be) the first complete mt genome (17,675 bp) for this genotype, being > 4 kb larger than any previously reported genome for this taxon.

This work stimulated us to scrutinise published mitochondrial genomic data sets of other flatworms, including the carcinogenic liver flukes *Clonorchis sinensis* (Chinese liver fluke), *Opisthorchis viverrini* (Southeast Asian liver fluke) and *Opisthorchis felineus* (cat liver fluke) [20–22]. There were indications of sequence complexity in mitochondrial non-coding regions and the potential for gaps in the published genomes. In the present study, our goal was to critically investigate the completeness of the mitochondrial genome of *C*. *sinensis* using Oxford Nanopore long-read sequencing technology (https://nanoporetech.com). We show the effectiveness of this technology to rapidly sequence the compete mitochondrial genome, irrespective of its length, nature or the structure of intergenic spacer region(s), and to enable the characterisation of large tandem-repeat regions within the mitochondrial genome of *C*. *sinensis*.

## Methods

### Parasite material

Adult worms of *C*. *sinensis* (*n* = 100) were collected in 2009 from Syrian golden hamsters (*Mesocricetus auratus*) experimentally infected with metacercariae isolated from naturally infected cyprinid fish (*Pseudorasbora parva*) originating from Jinju-si, Gyeongsangnam-do, the Republic of Korea, as described previously [23]. This work was conducted by one of the authors (W.-M.S.), in accordance with protocols approved by the animal ethics committee at Gyeongsang National University.

### Isolation of high molecular weight genomic DNA, library construction and sequencing

High quality DNA was isolated from the pool of 100 adults of *C*. *sinensis* using the Circulomics Tissue Kit (Circulomics, Baltimore, MD, USA). Subsequently, low molecular weight DNA was removed using the 5 kb- or 20 kb-Short Read Eliminator (SRE) kit (Circulomics, Baltimore, MD, USA). High molecular weight *C*. *sinensis* genomic DNA was used to construct rapid-sequencing (SQK-RAD004; Oxford Nanopore Technologies; 5 kb SRE) and ligation-sequencing genomic DNA libraries (SQK-LSK109; Oxford Nanopore Technologies; 5 and 20 kb SRE), according to the manufacturer’s instructions. The SQK-RAD004 (5 kb SRE) and SQK-LSK109 (5 kb SRE) libraries were sequenced using separate flow cells (R9.4.1; Oxford Nanopore Technologies). The flow cell used to sequence the SQK-LSK109 (5 kb SRE) library was washed using a Flow Cell Wash Kit (EXP-WSH003; Oxford Nanopore Technologies) and re-used to sequence the SQK-LSK109 (20 kb SRE) library. All genomic DNA libraries were sequenced (48 h) on the MinION sequencer (Oxford Nanopore Technologies). Following sequencing, bases were ‘called’ from raw FAST5 reads using the program Guppy v.3.1.5 (Oxford Nanopore Technologies) and stored in the FASTQ format [24].

### Assembly of the mitochondrial genome

The reads were mapped to the reference mitochondrial genome of a Korean isolate of *C*. *sinensis* (GenBank accession no. KY564177; [22]) using Minimap2 v.2.17-r941 [25]; mapped reads and their alignment positions were stored in the BAM format [26]. The mapped reads were extracted from the BAM file using SAMtools v.1.9 [26] and initially assembled using the program Canu v.2.0 [27]. Repeat sequences in the assembled mitochondrial genome were identified using the program repeat-match in the MUMmer package v.3.23 [28]. A library of identified repeat sequences and published mitochondrial protein genes of *C*. *sinensis* (GenBank accession no. KY564177; [22]) was used to assess the number of repeat units and completeness of the repeat region using the program RepeatMasker v.4.0.5 (http://www.repeatmasker.org). The final representative mitochondrial genome was assembled using reads that spanned the entire repetitive region encoding the commonest tandem-repeat unit frequency (± 1 repeat unit) and the program Canu. The non-repetitive region of the assembled genome was then polished with Pilon v.1.23 [29] using available Illumina short-read data [22]. Finally, all long-read data produced were mapped to the assembled mitochondrial genome using Minimap2, and coverage of the genome was determined using mpileup in the SAMtools package [26].

### Annotation of the mitochondrial genome and characterisation of the repeat region

The new assembly was compared with those of published mitochondrial genomes of *C*. *sinensis* (GenBank accession nos. KY564177, JF729304, JF729303 and FJ381664; [20–22]); subsequently, tRNA, rRNA and protein-encoding gene annotations were transferred to the assembled genome. The open reading frame (ORF) of each protein gene was verified using the program Geneious v.11.1.5 [30], employing the mitochondrial genetic code for echinoderms and flatworms ([7]; https://www.ncbi.nlm.nih.gov/Taxonomy/Utils/wprintgc.cgi#SG9). Secondary structures were predicted using the Vienna RNA Websuite ([31]; http://rna.tbi.univie.ac.at) and drawn using the tool Forna [32]. The complete mitochondrial genome sequence was deposited in the GenBank database under the accession no. MT607652; raw data are also available in the Sequence Read Archive (SRA) under the accession no. PRJNA386618.

## Results and discussion

### The mitochondrial genome of *C*. *sinensis* contains a tandem-repetitive region of > 4.5 kb

**Fig 1:**
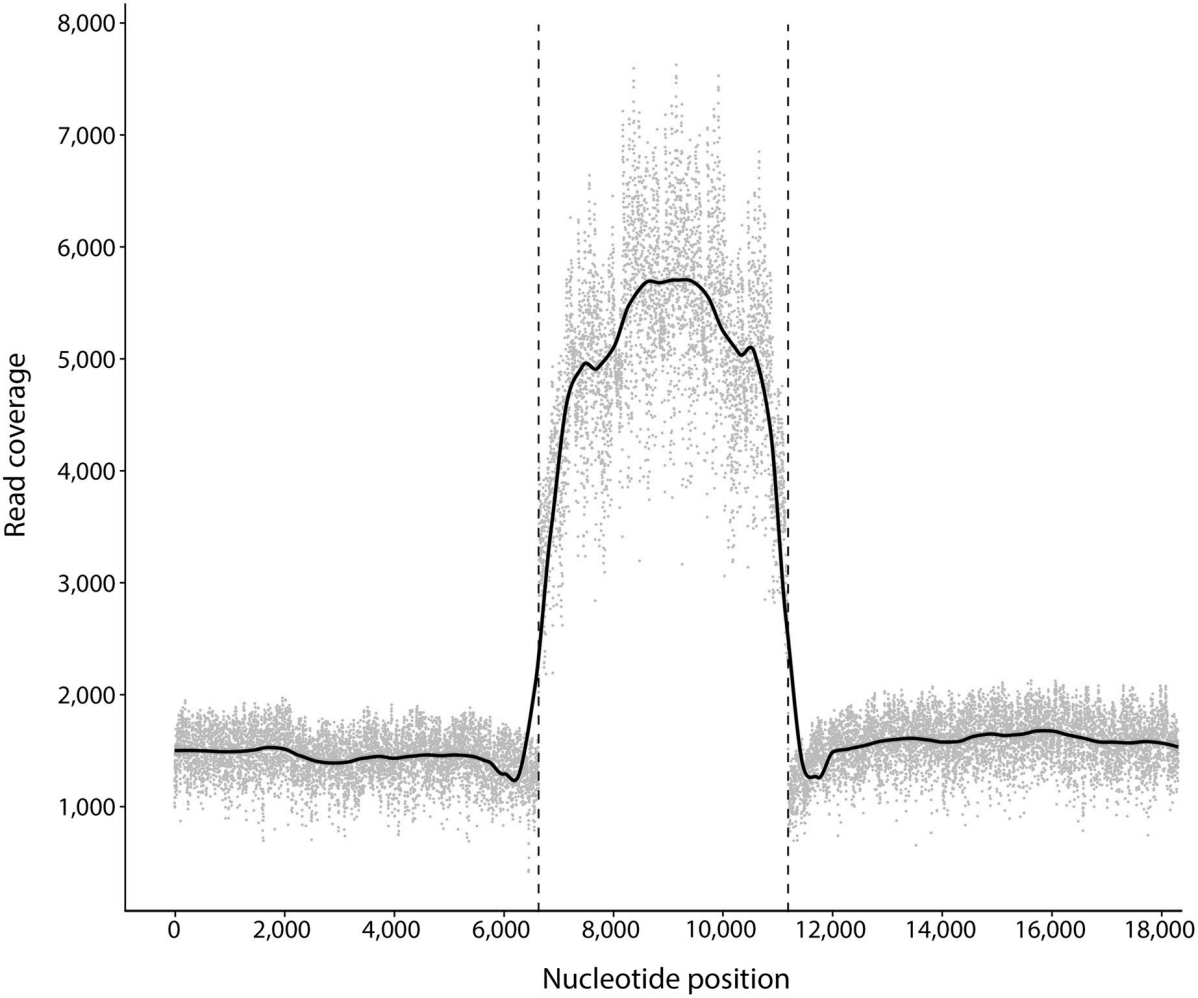
Coverage of long-reads (produced by Oxford Nanopore sequencing) across the mitochondrial genome (18,304 bp) of *Clonorchis sinensis*. The graph shows the depth of nucleotides at each position (grey dots) and the smoothed average of depth across the genome (solid black line). Dashed lines demarcate the start (position 6,640) and the end (11,188) of the long tandem-repeat region of 4,549 bp (Fig 2).

**Fig 2:**
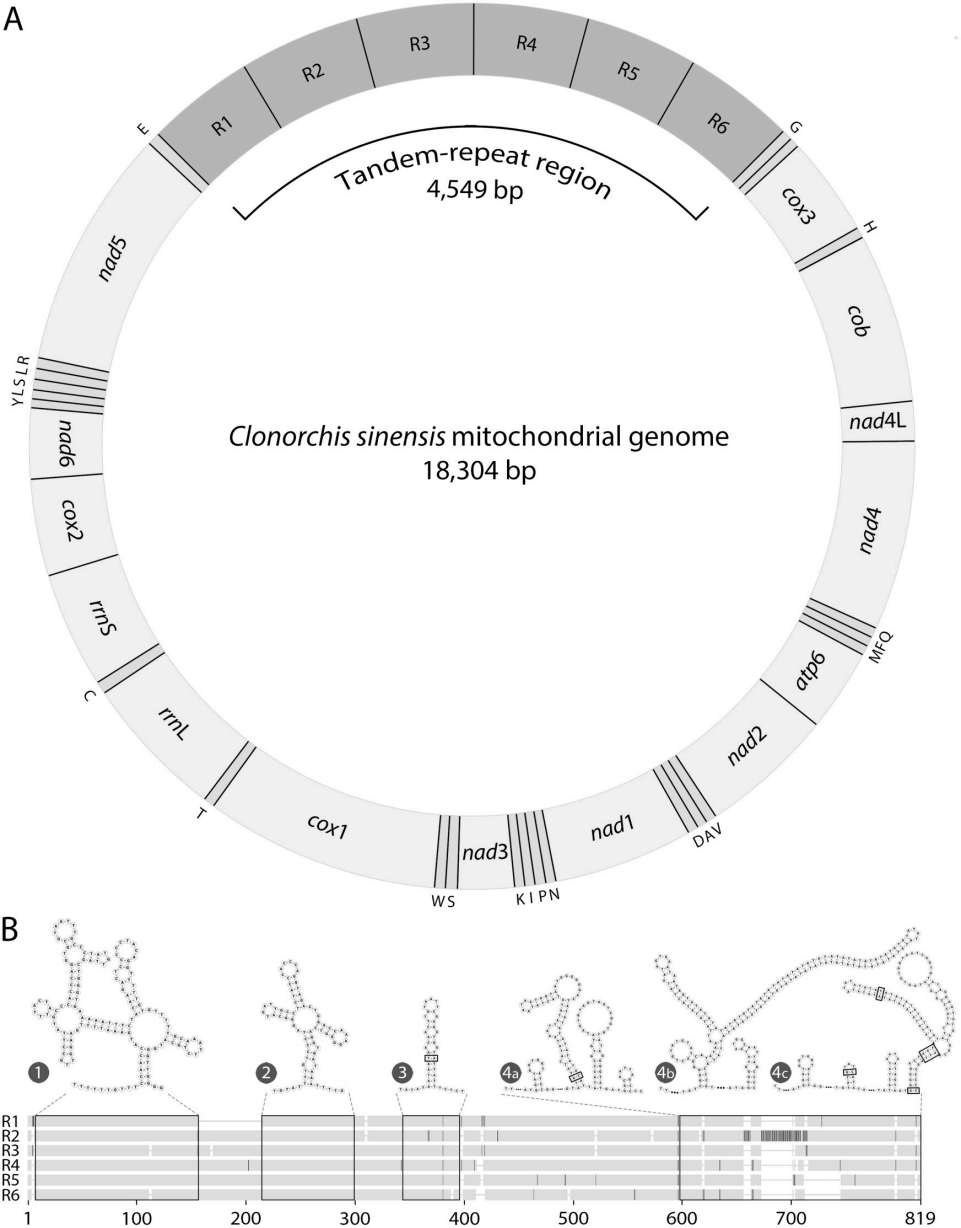
Complete, annotated mitochondrial genome of *Clonorchis sinensis*. (A) Schematic representation of the mitochondrial genome, including the newly-identified tandem-repeat region (4,549 bp); the 12 protein-encoding genes, 2 rRNAs and 22 tRNAs (designated by their one-letter amino acid abbreviations) are in accord with a published reference mitochondrial genome available in GenBank (accession no. KY564177; [22]). The short non-coding region is between tRNA-Gly (G) and *cox*3. (B) A schematic alignment of the tandem-repeat units (R1 to R6; bottom), showing nucleotide identities (light grey) or differences (dark grey) and regions predicted to assume structures (boxed in black). Secondary structures predicted for individual repeat units are indicated above the alignment; mis-matches in stems are indicated (boxed). Positions 598 to 819 include a variable TA-rich region and was predicted to fold into three distinct structures (4a to 4c).

From a total of 93,729 long-reads (equating to 310 Mb), we *de novo*-assembled a 18,304 bp mitochondrial genome for *C*. *sinensis* at high coverage (average: 2,381; median: 1,615; Fig 1), including a tandem-repetitive region (Fig 2). The initial assembly indicated variation in the number of repeats spanning this region, which likely related to sequence-length variation among individual worms used for the preparation of genomic DNA. In the first instance, we selected six repeats to represent this region. However, it was somewhat challenging to unequivocally assemble all sequences across this tandem-repeat region and to define its precise length. In order to establish the nature and extent of variation in the number and length of repeat sequences, we mapped all long-read data to the mitochondrial genome containing six tandem-repeats and showed a substantial increase in coverage (mean of 1,530 to 5,018; peak at 7,627) across this region (positions 6,640 to 11,188; Fig 1). Although mapping results identified reads containing more (*n* > 1,200) or less (*n* > 18,900) than six tandem-repeats, scrutinity of the data revealed 40 sequences (with 3 to 41 repeat units) that bridged the entirety of the tandem-repeat region and were flanked at each terminus by sequences that matched perfectly the expected genes (tRNA-Glu and *nad*5 at the 5′-end, and tRNA-Gly and *cox*3 at the 3′-end). Irrespective of this variation, reads with six tandem-repeats predominated. Hence, this number of repeats was selected to represent the mitochondrial genome of *C*. *sinensis* without considering the variation that exists among (or within) individual worms. In this representative mitochondrial genome, repeat units R1 to R6 (Fig 2) were 719–809 bp in length and had 91% identity upon pairwise comparison. Most differences related to length variation in TA- (69 to 138 bp) and GA-rich (26 to 35 bp) sequence tracts, although a 58 bp deletion occurred in a non-repetitive DNA segment (Fig 2). Parts of the repeat units were predicted to fold into secondary structures; some of these predicted structures were complex, with internal loops (≤ 10 bp) and multiple hairpins (stems: ≤ 39 bp; Fig 2).

### Variation in the tandem-repetitive region

Evidence of variation in the number of repeats spanning this long non-coding region raised a question about possible technical artefacts. However, because long, intact single-molecule DNA strands were sequenced here using Nanopore technology, such artefacts can be excluded (cf. [33]). Using this technology, we obtained long sequence reads for the entire long tandem-repetitive region, without the need for any read assembly. The use of direct library construction methods excludes artefacts, such as chimeric sequences, resulting from amplification [34–36]. Thus, reads that bridged the entire repeat region and had termini that matched respective flanking regions in the reference mitochondrial genome represented the tandem-repetitive region in *C*. *sinensis*.

Given that sequence/length variation in mitochondrial non-coding (e.g., control or intergenic) regions is commonly recorded among individuals of an animal species [37], we expected to find such variation in the tandem-repetitive region of *C*. *sinensis*, because we used a pool of *C*. *sinensis* adults to prepare genomic DNA for sequencing. Indeed, the mapping results revealed marked variation in sequence, length and repeat numbers as well as sequence coverage. This variation could be among individual worms, because DNA was isolated from 100 worms, but intraindividual or tissue-specific variability (i.e. heteroplasmy) cannot be excluded. Length variation in mitochondrial repeat regions, established using PacBio long-read sequence data, have been reported recently in other trematodes, such as *Paragonimus westermani* and *Schistosoma bovis* [18, 38], but the frequencies and patterns of occurrence within worm populations are unexplored. We believe that further sequencing is warranted to obtain complete (long) read data from individual worms of *C*. *sinensis* (preferably from disparate geographical areas) to gain an appreciation of the diversity in number and sequence of repeat elements within this non-coding region in *C*. *sinensis*. Although the origin(s) of such variation in flatworms is presently unknown, it might be the result of double-strand break repair or slipped-strand mispairing during replication [39, 40].

The identification in the sequence data set of long-reads containing > 6 repeat units that did not span the non-coding region (4.5 kb) suggested partial degradation of mitochondrial DNA in the total DNA sample—extracted from *C*. *sinensis* worms collected in 2009—used for nanopore-sequencing. Some degradation or nicking of repetitive DNA would be expected to occur in a sample stored frozen for such an extended period (11 years). However, it is also possible that secondary structural arrangements in repetitive elements (Fig 2) might have led to some nicking during sequencing, resulting in a proportion of incomplete sequences, which is plausible for long DNA strands.

### Overcoming the challenges of sequencing the tandem-repetitive region

The mitochondrial genomes of a range of flatworms (cestodes and trematodes) are known to harbour non-coding regions containing repetitive elements [2, 7]. Short and long non-coding regions appear to be characteristic of trematodes, although often partially sequenced using Sanger- or short-read sequencing methods [3, 7, 21]. The comparison of the present mitochondrial genome assembly with published mitochondrial genomes of *C*. *sinensis* revealed that the newly-characterised tandem-repeat region occurs between tRNA-Glu and tRNA-Gly, formerly estimated at 153–154 bp in size [20–22]. A short non-coding region between tRNA-Gly and *cox*3 equated to 67 bp, as reported previously (67 or 68 bp). All 12 protein-encoding genes, 22 tRNAs and two rRNAs had high sequence similarities (> 99.2%) to those in published mitochondrial genomes and occurred in the same order. However, there is clear evidence [17, 18, 38] that conventional sequencing methods are not suited to the sequencing of long non-coding regions in mitochondrial genomes. This obstacle has been overcome through the use of nanopore-sequencing, which bodes well for future mitochondrial genome investigations.

### Speculating about the role(s) of non-coding elements in the mitochondrial genome

Although the functions of long non-coding elements in the mitochondrial genome of parasitic flatworms are unexplored, they are hypothesised to be ‘control’ regions, which initiate replication and transcription [7, 41–44]. In bilaterian animals, the control region is typically ~ 1 kb in size [45–49] and often contains short repeat elements, predicted to fold into secondary structures [37]. Although significant deviations from a ‘typical’ animal mitochondrial genome exist [50] and duplications of control regions are known to occur [51–55], expansive repetitive non-coding regions with substantial size variation within a species seem to be unusual. For parasitic flatworms, we propose that each tandemly-repeated unit represents a distinct control region possibly enhancing replication and transcription efficiency [17]. Multiple control regions within the mitochondrial genome might provide an advantage in terms of being able to adapt cellular energy production and metabolism during particular life-cycle phases while under strong selective pressure in different environments, both outside of or within a host animal (e.g., O_2_, pH, salinity, temperature, light, osmotic pressure and/or nutrient accessibility).

Efficient replication might also limit the detrimental effect of extreme environments on mitochondrial DNA integrity. A plethora of internal and external agents (e.g., reactive oxygen species, metabolites, radiation, environmental chemicals and toxins) are known to cause DNA damage such as mutations and lesions, of which double-strand breaks (DSBs) are particularly harmful [56–58]. Although animal DNA is constantly exposed to such stressors, it could be proposed that many organisms, such as parasitic helminths, inhabit particularly inhospitable environments that cause chronic damage to mitochondrial DNA and that unique strategies might have evolved to achieve efficient genome maintenance and ensure cellular viability. Conditions potentially disrupting the mitochondrial DNA integrity of *C*. *sinensis* could include exposure to toxic bile salts and acids and/or desiccation, which have been shown to cause DNA anomalies such as DSBs in some microbe and metazoan species [59–66]. In response to this stress, replication of the mitochondrial genome might need to be highly efficient, in order to have a high number of genomes in the cell at any one time. This might avoid harmful mutations in the mitochondrial genome by increasing the number of template molecules in each cell, required to repair DNA in the least error-prone way [58, 67, 68]. A large number of genomes might act also as a ‘buffer’ in the cell—even if some get damaged, many functionally intact genomes will be present, ensuring that replication and transcription of mitochondrial genes are not disrupted within the cell. Whether selection acts upon the size of the repeat region in the mitochondrial genome of *C*. *sinensis*, or whether repeat expansions and contractions represent stochastic events, such as errors during DNA repair (e.g., [40]), warrants investigation. Future work might explore whether the repetitive region might function as an ‘origin of replication’ using a combination of two-dimensional neutral agarose gel electrophoresis and electron microscopy techniques [69].

### Concluding remarks

The first characterisation of a novel tandem-repetitive region (> 4.5 kb) in *C*. *sinensis* and variation in the sequence and number of repeat elements within this region raise questions about (i) the functional role(s) of this region within cells and mitochondria; (ii) the origin of such variation and whether it occurs within cells or tissues within individual worms, or among worms; and (iii) what impact such variation has on mitochondrial, nuclear and/or cellular functions. In our opinion, these research questions would be interesting to pursue in the near future.
